# Supplementary material for: Preparation of a Novel Cellulose–Styrene Copolymer Adsorbent and Its Adsorption of Nitrobenzene from Aqueous Solutions
Source: Polymers (Basel). 2021 Feb 18;13(4):609. doi: 10.3390/polym13040609 (PMC7922655; doi:10.3390/polym13040609)
Supplement: Supplementary file 1 [file polymers-13-00609-s001.pdf]

Supplementary Materials

Preparation of a Novel Cellulose–Styrene Copolymer Adsorbent and Its Adsorption of Nitrobenzene from Aqueous Solutions

Guifang Yang [1,2], Na Lin [1], Yuan Li [1], Xiaoxia Ye [1], Yifan Liu [1,2], Yuancai Lv [1], Chunxiang Lin [1] and Minghua Liu [1,2,*]

[1] College of Chemical Engineering, Fuzhou University, Fuzhou 350116, China; M150410005@fzu.edu.cn (G.Y.); N190627042@fzu.edu.cn (N.L.); N180627037@fzu.edu.cn (Y.Li); yexiaoxia@fzu.edu.cn (X.Y.); yfanym@fzu.edu.cn (Y.Liu); yclv@fzu.edu.cn (Y.Lv); lcx2010@fzu.edu.cn (C.L.)

[2] Fujian Provincial Engineering Research Center of Rural Waste Recycling Technology, College of Environment & Resources, Fuzhou University, Fuzhou 350116, China

* Correspondence: mhliu2000@fzu.edu.cn; +86 133-0502-2089

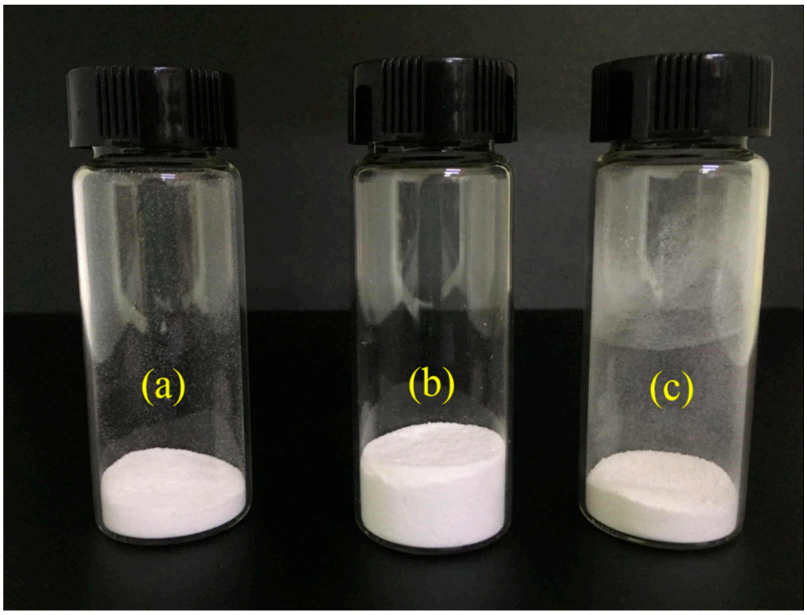

Fig. S1. 1g of origin cellulose (a), cellulose-OH (b) and cellulose-St (c)

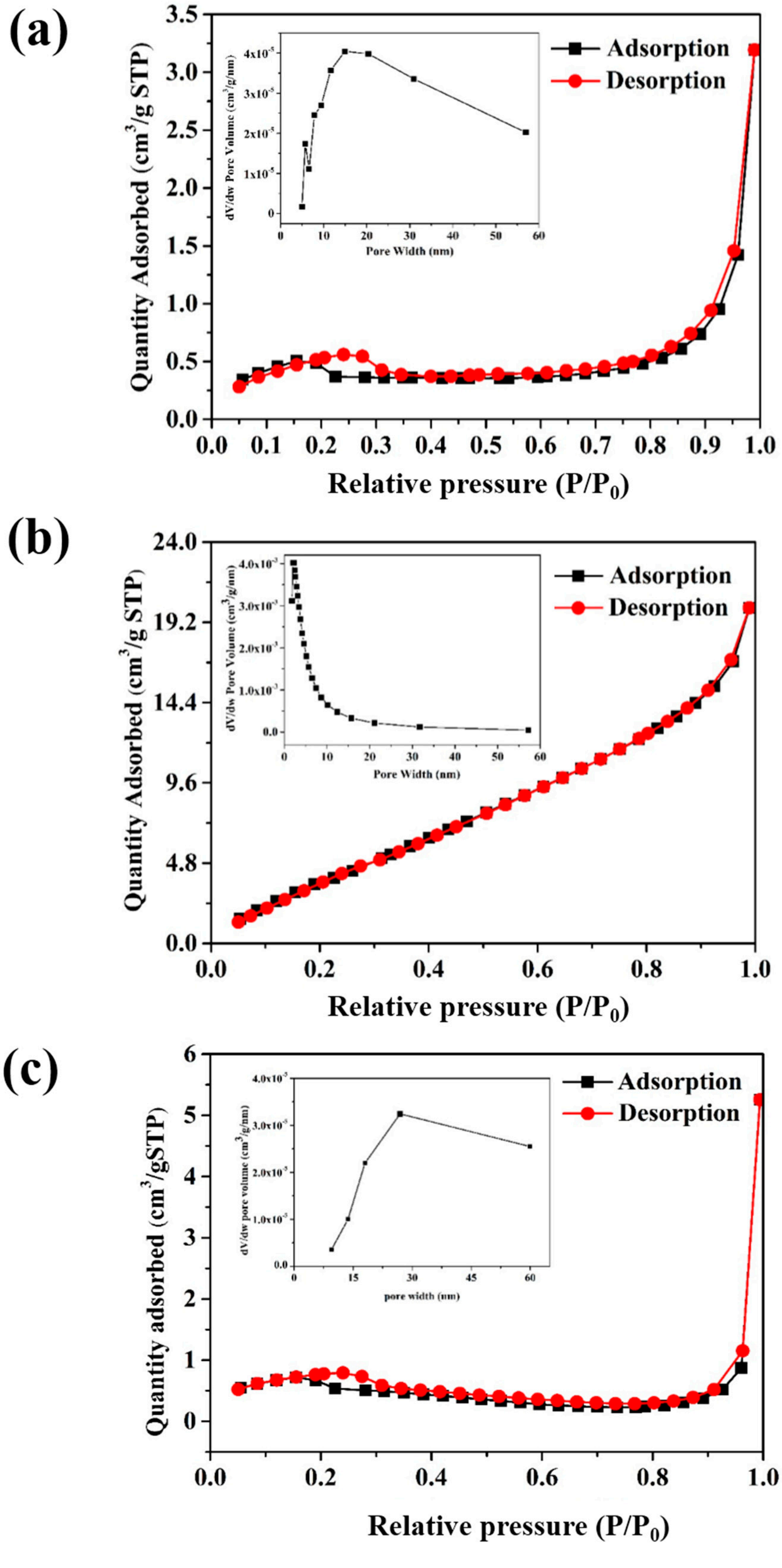

Fig. S2. The N_2 adsorption-desorption isotherms and the pore size distribution of cellulose (a), cellulose-OH (b) and cellulose-St (c)

Table 1. Experimental data derived from BET analysis of cellulose, cellulose-OH and cellulose-St.

Sample	BET Surface Area(m^2/g)	Adsorption average pore size (nm)	Pore Volume (cm^3/g)
Cellulose	1.0441	18.92506	0.00494
Cellulose-OH	19.3288	6.42269	0.031036
Cellulose-St	1.4630	22.87448	0.008127

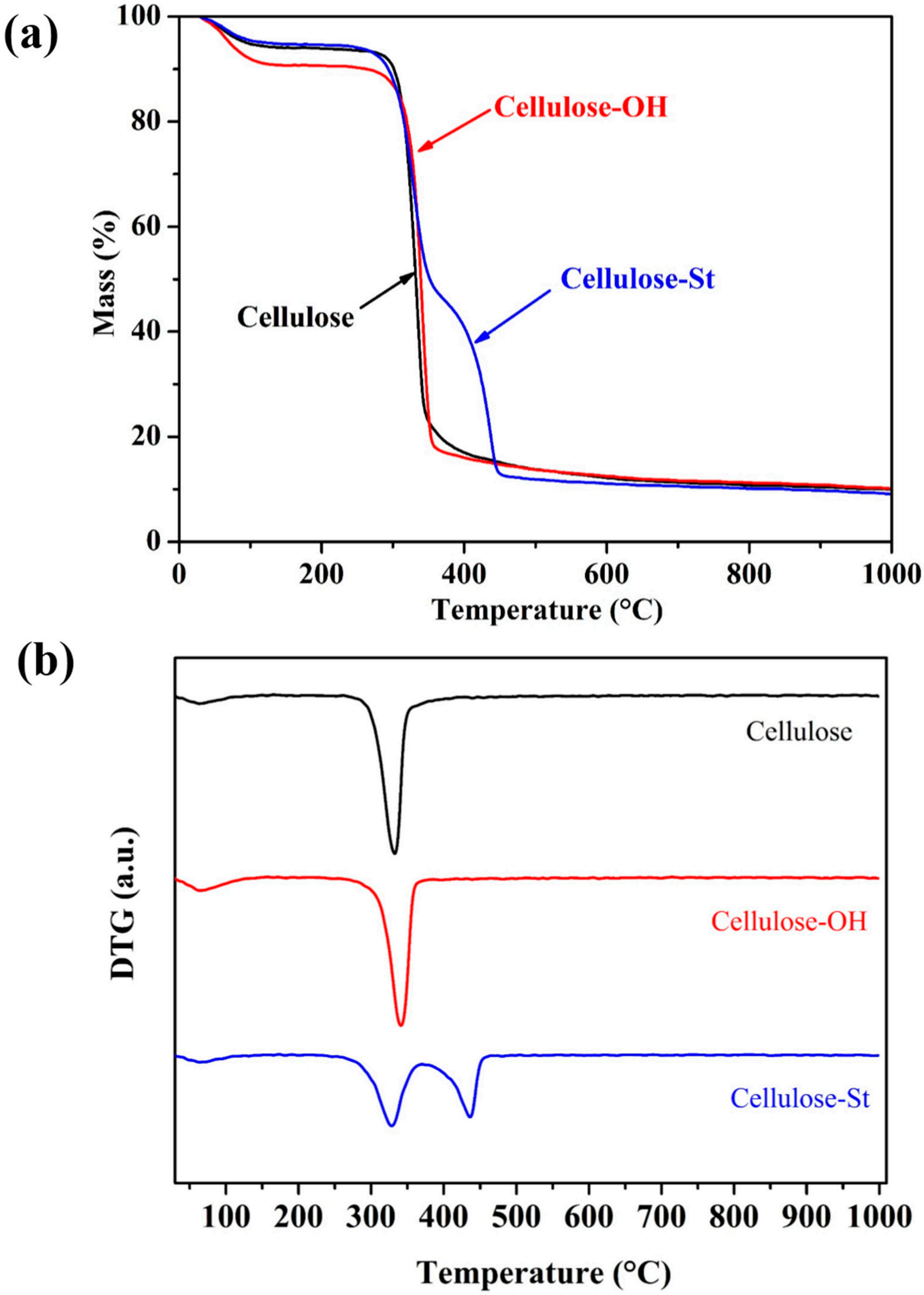

Figure 3. TGA (a) and derivative TGA (DTG) (b) of cellulose, cellulose-OH and cellulose-St.

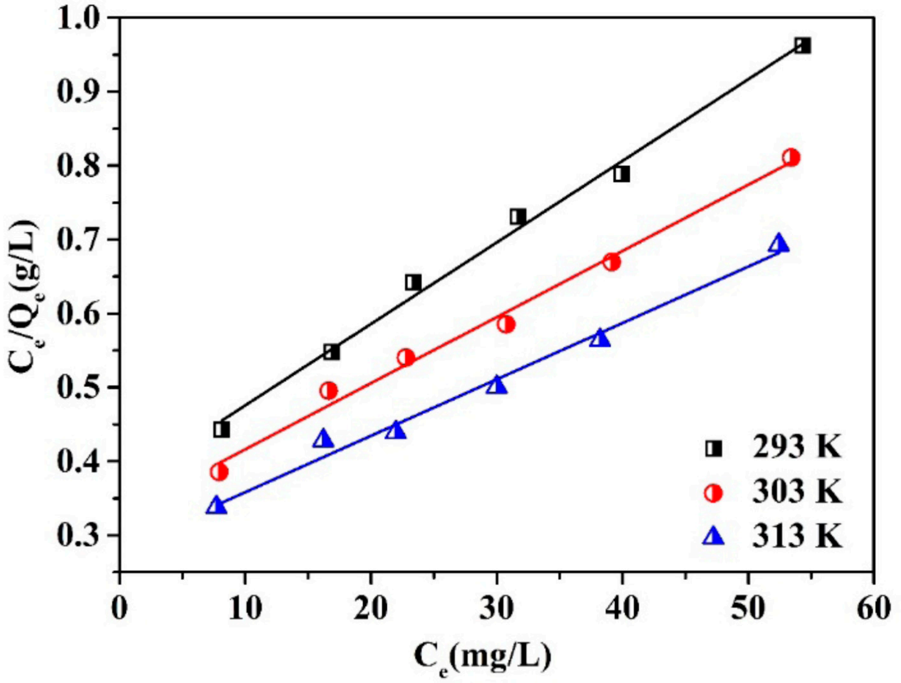

Fig. S4 Linear fitting with Langmuir model at different temperature for NB adsorption over cellulose-St

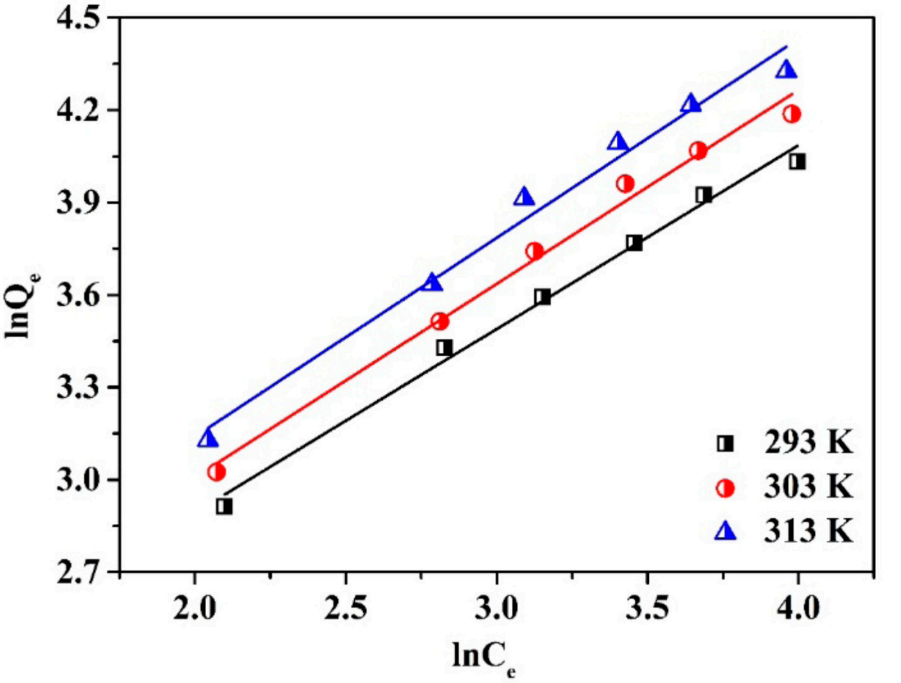

Fig. S5 Linear fitting with Freundlich model at different temperature for NB adsorption over cellulose-St

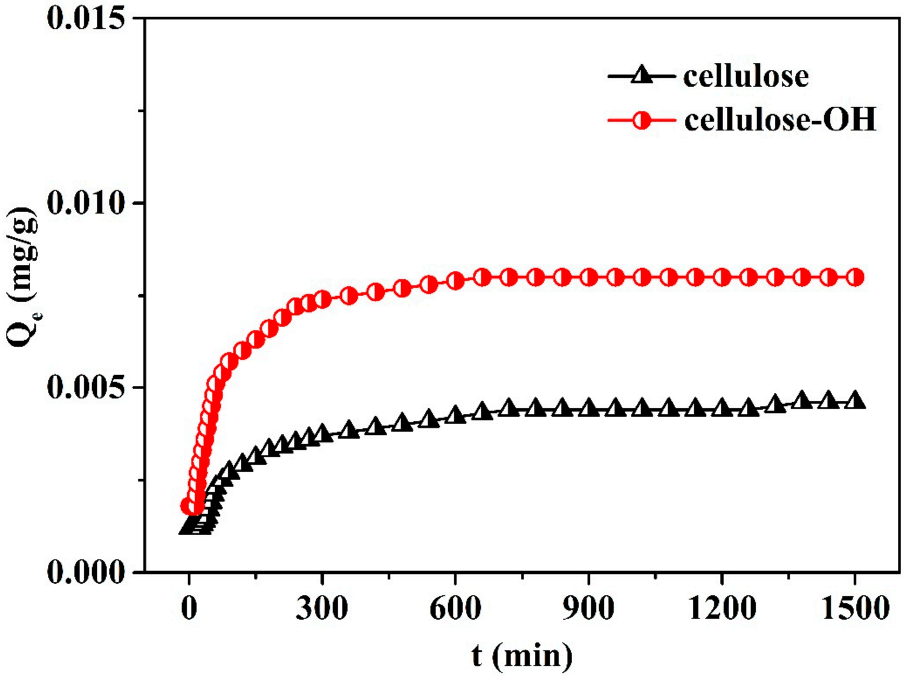

Fig. S6. Adsorption of NB on cellulose and cellulose-OH (Temperature: 25 °C; initial NB concentration: 10 mg/L; amount of cellulose or cellulose-OH: 10 mg; volume of NB solution: 50 mL.)

Table S2 Adsorption isotherm parameters for the adsorption of NB on cellulose-St at different temperatures

Temperature	Langmuir			Freundlich		
(K)	Q_m (mg/g)	K_L	R^2	K_F	1/n	R^2
293	90.66	0.0302	0.992	5.48	0.596	0.998
303	111.73	0.0274	0.989	5.75	0.629	0.998
313	131.06	0.0271	0.984	6.35	0.646	0.990

Table S3 Nitrobenzene adsorption performance over reported activated carbon materials

Absorbents	pH adaption	Adsorption capacity (mg/g)	Reusability	References
Commercial granular activated carbon	-	87	-	[1]
Activated carbon from rice husk	4-10	446	-	[2]

Acidic oxygen functionalized activated carbon	-	42	-	[3]
Activated carbon from wood	-	238	-	[4]
Activated carbons prepared from vegetable waste	2-12	476	58.7% after 4 runs	[5]
Activated carbons prepared from vegetable waste	2-12	490	62.8% after 4 runs	[5]
Magnetically separable porous carbon microspheres	-	97	84% after 5 runs	[6]
cellulose-styrene copolymer	2-12	131	90.1% after 10 runs	This work

References

1. Jadhav, A.J. and Srivastava, V.C., 2013. Adsorbed solution theory based modeling of binary adsorption of nitrobenzene, aniline and phenol onto granulated activated carbon. Chemical engineering journal, 229, pp.450-459.
2. Dasgupta, A., Matos, J., Muramatsu, H., Ono, Y., Gonzalez, V., Liu, H., Rotella, C., Fujisawa, K., Cruz-Silva, R., Hashimoto, Y. and Endo, M., 2018. Nanostructured carbon materials for enhanced nitrobenzene adsorption: physical vs. chemical surface properties. Carbon, 139, pp.833-844.
3. Kato, Y., Machida, M. and Tatsumoto, H., 2008. Inhibition of nitrobenzene adsorption by water cluster formation at acidic oxygen functional groups on activated carbon. Journal of colloid and interface science, 322(2), pp.394-398.
4. Dai, Y., Mihara, Y., Tanaka, S., Watanabe, K. and Terui, N., 2010. Nitrobenzene-adsorption capacity of carbon materials released during the combustion of woody biomass. Journal of hazardous materials, 174(1-3), pp.776-781.
5. Kecira, Z., Benturki, O., Benturki, A., Daoud, M. and Girods, P., 2020. High adsorption capacity

of nitrobenzene from aqueous solution using activated carbons prepared from vegetable waste. Environmental Progress & Sustainable Energy, 39(6), p.e13463.

6. Zhu, Y., Zhang, L., Schappacher, F.M., Pöttgen, R., Shi, J. and Kaskel, S., 2008. Synthesis of magnetically separable porous carbon microspheres and their adsorption properties of phenol and nitrobenzene from aqueous solution. The Journal of Physical Chemistry C, 112(23), pp.8623-8628.
